# Influence of aging on hyaluronic acid concentration in the vocal folds of female rats

**DOI:** 10.1590/S1808-86942012000300003

**Published:** 2015-10-14

**Authors:** Hugo Valter Lisboa Ramos, Luciano Rodrigues Neves, João Roberto M. Martins, Helena B. Nader, Paulo Pontes

**Affiliations:** a PhD – Federal University of São Paulo (Attending Physician at Hospital São Paulo).; b PhD – Federal University of São Paulo (Attending Physician at Hospital São Paulo, Department of Otorhinolaryngology and Head and Neck Surgery – Federal University of São Paulo).; c PhD (Professor and Associate Researcher – Department of Biochemistry from the Molecular Biology Division and Department of Medicine – Endocrinology Program – Federal University of São Paulo – UNIFESP.; d Post-Doctoral Researcher (Full Professor – Department of Biochemistry, Molecular Biology Division, Federal University of São Paulo).; e Senior Associate Professor (Full Professor of Otorhinolaryngology and Head and Neck Surgery; Director of the São Paulo Campus - Vila Clementino - UNIFESP).

**Keywords:** aging, larynx, vocal cords, rats, hyaluronic acid

## Abstract

The vibration of the vocal fold lamina propria is an important factor involved in vocal production and aging may change the amount of hyaluronic acid in the vocal fold leading to dysphonia.

**Aims:**

This study compares the concentration of hyaluronic acid in vocal folds of aged and young female rats. Study design: experimental.

**Materials and Methods:**

We used the vocal cords of 13 female rats divided into two groups: five aged rats and eight young ones. The tissue concentration of hyaluronic acid was determined using the fluorimetric method with the hyaluronic acid binding-protein coated on plates of enzyme-linked immunosorbent assay (ELISA), conjugated with biotin. Europium-labeled streptavidin was added and, after europium release with the use of enhancement solution, the final fluorescence was measured in a fluorometer.

**Results:**

We found the following concentrations of hyaluronic acid in vocal fold according to the group: 581.7 ng/mg in old female rats and 1275.6 ng/ mg in young female rats. Statistical analysis showed differences between groups.

**Conclusions:**

The vocal folds of old female rats have a lower concentration of hyaluronic acid when compared to such concentration on the vocal folds of young female rats.

## INTRODUCTION

Since the first histology studies involving the vocal folds (VF)^1^, which showed the close relationship between sound production physiology and the lamina propria (LP) - structure of the folds, this region has triggered the attention of laryngologists all over the world.

The VF has a LP rich in glycosaminoglycans (GAG), which are the interstitial proteins responsible for the filling and viscosity of these tissues.

Hyaluronic acid (HA), one of the main LP GAG, has a high density of negative charges in its structure. This makes it highly hydrophilic, making it the main responsible for viscosity, fluidity and tissue filling^2^, ^3^, ^4^.

Numerous types of disorders can change the amount of HA in the VF and compromise phonation quality - and aging is one of the most prevalent. Glottic characteristics indicative of presbylarynx, such as an arched VF and vocal process prominence, have been described by many authors^5^, ^6^, ^7^ and suggest VF swelling reduction, with a possible reduction in HA concentration.

Other authors describe the effects of aging on the fibrous proteins, showing an atrophy of the elastic fibers and increase in the density of VF collagen fibers^8^, ^9^.

Although proven a reduction in the gene expression which code vocal fold HA synthase on the vocal fold of elderly rats^10^, the attempts to quantify the differences in HA quantities associated with aging were not well succeeded and require more specific methodological studies^11^, ^12^. Tissue coloring methods which simultaneously identify various types of GAGs are, often times, wrongly utilized to assess HA quantity.

The goal of the present study is to compare the concentration of hyaluronic acid in the VF of female aged rats.

## MATERIALS AND METHODS

This research Project was analyzed and approved by our Ethics Committee, and this study is experimental.

We used the VFs of 13 female Wistar-sp rats, weighing about 250 grams. We considered as aged, those rats with ages near 24 months.

We included eight young and eight old female rats, not pregnant and not breastfeeding, without any prior use of medication and under *ad libitum* feeding. The larynx was removed around 2 minutes after the slaughter and was preserved in eppendorf micro tubes with acetone.

### Sample preparation

Both vocal folds from each female rat were removed from the larynx and fragmented in small particles. The tissue was weighed after centrifuging and drying, and it was then submitted to protein digestion under protease (alkaline protease maxatase; Biocon do Brasil Industrial, Rio de Janeiro, Brazil). After protease inactivation by boiling, the samples were centrifuged and the floating layer was harvested for HA quantification.

### HA Quantification

In order to measure the HA, we used the fluorometric method with the hyaluronic acid ligand protein (HALP)^13^. HALP is isolated from bovine cartilage and involves the globular region of a proteoglycan called agrecan. In our study, it was immobilized in *enzyme-linked immunosorbent assay* (ELISA) plates, similar to a ligation antibody, and also as a biotin-tandem probe, working as a marked secondary antibody.

The ELISA plates were coated with HALP and incubated with samples of a hyaluronic acid standard-solution (000-500 mg/l), to serve as a fluorescein standard curve, and with the sample solution obtained from the VF, in tripled.

Afterwards, the ELISA plates were washed and the biotin-tandem HALP was added. After a new washing process, we added europium-marked streptavidin. After europium is released from streptavidin with the use of an *enhancement* solution, the final fluorescence was measured in a fluorometer.

The data was interpreted and analyzed by means of a Mann-Whitney non-parametric test.

## RESULTS

Table 1 shows the mean age of the rats, in accordance with the comparison groups, followed by the standard deviation of each group. One can notice the major age difference between the young and old rats.

**Table 1:** Rats' ages according to the groups.

	Older	Young
	rats	rats
N	5	8
Mean age (days)	722.2	135.5
Standard deviation	28.1	35.0

The group of young rats had a mean age of 135.5 days and it is formed by sexually mature adults. On the other hand, the group of old rats had a mean age of almost two years.

Table 2 shows the HA concentration from each sample according to the groups.

**Table 2:** HA concentration in ng/mg of dry weight, in the vocal folds of rats, according to their assigned groups.

	Older rats	Young rats
	HA	HA
1	527.2	670.5
2	762.0	801.0
3	755.4	1039.4
4	394.1	1474.7
5	469.7	1577.5
6	-	1792.0
7	-	1806.7
8	-	1043.4
Mean	581.67	1275.64
Standard Deviation	168.39	444.11

HA – hyaluronic acid.

In order to compare the groups, we employed the Mann-Whitney non-parametric test (Table 3). We may, then, notice a significant difference in HA concentration between the groups of young and older rats. The older rats have a lower concentration of HA in their VF.

**Table 3:** Mann-Whitney test, employed to compare the groups.

	Mann-Whitney test (*p* value)
Older female x young female	0.006

## DISCUSSION

One of the first studies to detect GAGs on VFs used immunohistochemistry to identify the HA receptor, since the antibodies are not capable of directly ligating to the HA^2^.

Other authors^11^ have studied hyaluronic acid with the Muller-Mowry Colloidal Iron dye, which identifies acid mucopolysaccharides and, therefore, dyes many types of GAGs. Quantification happened by colorimetric comparison of slides before and after treatment with testicular hyaluronidase, which digests hyaluronic acid, but it also digests the chondroitin sulphate GAG. Using a sample of 10 men and eight women and with one unspecific method to identify and quantify HA, this study was also the first to make gender comparisons, showing a greater concentration of HA in men.

Also using the Muller-Mowry Colloidal Iron dye, other authors^12^ reported the presence of HA in human VFs, and they compared age and gender. They used nine adult male larynxes (between 34 and 52 years), seven adult female larynxes (between 21 and 41 years), four elderly male larynxes (between 65 and 77 years) and five elderly female larynxes (between 65 and 82 years). The authors, then concluded, in relation to gender that, the women had less HA in the VF surface layer. Nevertheless, in deeper layers, the amount of HA was greater than that in men. As far as age is concerned, the colorimetric difference was not significant.

Another unspecific tissue dying method, which simultaneously dyes different types of GAGs is the Alcian Blue. Some authors have utilized this dying method, without digestion by hyaluronidase, to identify age-related HA changes to the VF^14^. These authors utilized eight young and eight older rats and found lower values in the group of older rats.

Differently from previous studies, ours utilized a specific quantification method for HA, which provides its concentration in nanograms per milligram of dry weight^13^.

Other authors utilized this same fluorescence method for the HA quantification in the human vocal fold^15^. Nonetheless, they did not study possible differences in the HA concentration as far as age is concerned.

Our study utilized the vocal folds of rats and, because of the diminutive size of the rat's VF and the impossibility of separating the lamina propria from the adjacent muscles, the entire vocal fold depth was measured. Therefore, our results reflect HA concentration in the lamina propria and in the thyroarytenoid muscle together.

Most of the studies which use human VF as a study material remove the larynx many hours after death. However, in our study the mean time between the death and tissue fixation was 2 minutes. Considering that GAGs are substances which degrade fast, a fast VF fixation contributes to enhance the accuracy of our results.

Airway manipulation before death and the time elapsed between the death and the vocal fold fixation are factors which have a high potential to cause bias. The possibility of avoiding these errors reinforces the aforementioned advantages of using mice as animal models to study the vocal folds^16^. Moreover, the capacity of comparing controlled genetic lineages with a lower variability between the individuals makes comparison groups more homogeneous and the results more reliable.

It is very difficult to obtain older rats, because of their high mortality. Some authors have shown that Wistar rats live the longest – 34 months, and reach old age at around 24 months, a time when their mortality rates reach 50%^17^, ^18^. This difficulty in obtaining samples of older rats was the very reason why we did not have male rats in our study.

It is known that a deep understanding of the LP of vocal folds requires understanding the different functions of each one of the substances involved in VF vibration. Thus, the hyaluronic acid, as one of the main components of the extracellular matrix, is fundamentally important for the vocal fold mucosal wave laxity and for an optimal vocal quality.

Therefore, we believe that our study may contribute with important information. With an accurate method which yields the tissue concentration of HA in relation to the animal's dry weight, our study showed that aging reduces the amount of HA in the VF of female rats.

## CONCLUSION

The results enable us to conclude that the vocal fold or older female rats have a lower concentration of hyaluronic acid when compared to those from young female rats.
